# Violence towards health care workers in a Public Health Care Facility in Italy: a repeated cross-sectional study

**DOI:** 10.1186/1472-6963-12-108

**Published:** 2012-05-02

**Authors:** Nicola Magnavita, Tarja Heponiemi

**Affiliations:** 1 Institute of Occupational Medicine Università Cattolica del Sacro Cuore, Largo Gemelli 8, 00168, Roma, Italy; 2 National Institute for Health and Welfare, Helsinki, Finland

**Keywords:** Aggression, Violence at the workplace, Health care workers, Post-traumatic stress, Organizational justice, Work stress, Social support, Job control, Demand, Psychological disorders.

## Abstract

**Background:**

Violence at work is one of the major concerns in health care activities. The aim of this study was to identify the prevalence of physical and non-physical violence in a general health care facility in Italy and to assess the relationship between violence and psychosocial factors, thereby providing a basis for appropriate intervention.

**Methods:**

All health care workers from a public health care facility were invited to complete a questionnaire containing questions on workplace violence. Three questionnaire-based cross-sectional surveys were conducted. The response rate was 75 % in 2005, 71 % in 2007, and 94 % in 2009. The 2009 questionnaire contained the VIF (Violent Incident Form) for reporting violent incidents, the DCS (demand/control/support) model for job strain, the Colquitt 20 item questionnaire for perceived organizational justice, and the GHQ-12 General Health Questionnaire for the assessment of mental health.

**Results:**

One out of ten workers reported physical assault, and one out of three exposure to non-physical violence in the workplace in the previous year. Nurses and physicians were the most exposed occupational categories, whereas the psychiatric and emergency departments were the services at greatest risk of violence. Workers exposed to non-physical violence were subject to high job strain, low support, low perceived organizational justice, and high psychological distress.

**Conclusion:**

Our study shows that health care workers in an Italian local health care facility are exposed to violence. Workplace violence was associated with high demand and psychological disorders, while job control, social support and organizational justice were protective factors.

## Background

The problem of aggression towards health care staff is global and on the increase [1,2]. It is difficult to gage the extent of the problem since under-reporting of violent incidents is common [3] and is probably influenced by social roles or cultural factors [4]. In fact, workers often expect violence to be part of their job [5]. However, it is also possible that people who are under considerable stress, exaggerate their responses in an effort to provide suggestions for increasing safety at work. Annual rates of physical aggression against health care workers range from a low 3.1 % [6], to a prevailing 11-25 % [7-12], or even higher (35-71 %) levels [13-16]. Non-physical aggression rates are even more difficult to evaluate; assessments range from 38 % to 90 % in a one-year period [6,7,9,10,14-18]. Studies suggest that even if patients, their relatives and friends are the main perpetrators, much of the violence encountered by health care workers is from co-workers and managers [19]. Experiences of non-physical and physical violence among health care workers are associated with decreased job satisfaction, increased occupational strain, and poor patient care outcomes [14,20,21]. In addition, workplace violence has a negative impact upon health care workers’ commitment to their facility [22]. Moreover, the consequences for the patients and the entire facility are serious since the perception of violence is related to adverse patient outcomes on account of the negative quality of care and treatment [10].

It is difficult to analyze the literature on health care violence because of differences in definitions and methods [23]. There are many different definitions of “violence” and even more ways of collecting data, that range from self-reports to secondary analysis of workers' compensation claims. Although many studies have examined psychiatric settings [12,13] or emergency departments [24,25], few have considered public health care facilities and, more specifically, variations in professions and locations [26]. Some studies focus solely on nurses and do not take into consideration other health care workers. Other studies have been carried out by external researchers with a presumably limited knowledge of the workplace. Data have been collected at a single point in time, in many cases with a low response rate (e.g.: 24 % [7], 25 % [27], 33 % [17], 36 % [8], 39 % [15]). This lack of knowledge hampers preventive measures. Furthermore, some studies refer only to patient and visitor aggressive behavior [16] and fail to consider worker-on-worker (“internal”) violence, although it has been observed that rates for worker-on-worker violence may sometimes exceed rates for patient-to-worker violence [27]. Co-worker violence threatens the well-being of hospital employees and should be regularly tracked with other forms of workplace aggression [26].

Another issue which needs to be clarified is the relationship between violence and organizational justice, a psychosocial variable which has emerged in recent years as a determinant of workers’ health [28]. A previous study indicated that verbal violence is associated with low organizational justice in nursing students [19]. Psychosocial correlates of violence, such as mental health [29], job strain, and organizational justice [28] are particularly important: firstly, as they demonstrate that workplace violence is a significant occupational hazard, and secondly, because preventive programs should primarily focus on these issues.

The Italian Ministry of Health recently issued a Recommendation specifically calling for the prevention of violence in health care facilities [30]. However, this recommendation has been disregarded, and Italian health care institutions still lack policies, strategies and administrative or behavioral provisions to counteract workplace violence.

The purpose of this study is: (1) to evaluate the prevalence (one-year incidence) of physical and non-physical violence in an Italian local health care facility from 2005 to 2009; (2) to study the association between violence and psychosocial variables in order to promote the adoption of preventive measures. To overcome the aforementioned epidemiological problems we chose to administer questionnaires during periodical medical examinations at the workplace.

## Materials and methods

### Subjects

District I of the Local Health Care Facility RMF (Civitavecchia) includes one hospital and some local health services. This study included all workers undergoing workplace medical examination who had been employed in that facility for at least one year (676 subjects). Periodical medical examination is compulsory in Italy for all employees exposed to occupational risks (e.g. chemical, biological, physical and organizational/psychosocial hazards). The physician responsible for the medical surveillance of workers (NM) was not on the company staff, but was a university employee who had been working as a consultant for the facility since 1999 and had therefore gained the workers’ confidence. For many years the workers had been accustomed to completing questionnaires while waiting for medical examination so they were aware that the questionnaires were anonymous and that the results would be used in their interest. Most of the workers chose to complete the questionnaire, although participation was not obligatory. In order to avoid coercion, workers completed questionnaires in a separate room while waiting for medical examination; the occupational physician was unaware of the workers’ decision. In order to guarantee privacy and anonymity, demographics were limited to gender, age, job and department. The Ethics Committee of the Catholic University School of Medicine approved the study design.

The present data consist of three cross-sectional surveys conducted in 2005, 2007 and 2009. We chose a two-year period for our surveys in order to avoid duplicate reporting. In the 2005 and 2007 surveys, workers were invited to report their experience of violence at the workplace over the previous 12-month period, making a distinction between physical and non-physical aggression. In addition to single-item questions on the occurrence of physical and non-physical violence, in 2009 the questionnaire included domains on mental health, job stress and organizational justice.

Respondents were allocated to groups according to the department in which they were employed at that time.

### Questionnaire

Physical aggression is defined as forceful, hostile or aggressive behavior which may or may not cause harm. A threat refers to the menace of causing harm. Verbal (non-physical) aggression is defined as any annoying or unpleasant act (words, attitudes, actions) that creates a hostile work environment. Harassment is defined as behavior characterized by insistent requests, messages, phone calls or other unsolicited contact that may cause annoyance, worry or fear.

The characteristics of incidents were studied using the Italian version of the VIF (Violent Incident Form), a validated questionnaire proposed by Arnetz for the registration of violent incidents in the health care workplace [31] and previously used in other Italian studies [19,32-36]. The VIF consists of 11 clear-cut questions with binary (yes/no) responses for describing a specific incident of violent or harassing behavior directed toward a staff member. It includes domains about the perpetrator (origin, sex, age, status), the activity that preceded the incident, the type of assault, the action taken by the victim and the consequences of the incident. Consequences include fear, anger, distress, anxiety, humiliation, guilt, disappointment, helplessness, absence of reaction, physical injury and others, such as a desire for revenge, a feeling of being in the wrong, an intention to quit the place of work or to change personal behavior. In the questionnaire it was possible to distinguish between external and internal violence, i.e. between aggression perpetrated by patients, their relatives or friends, and aggression on the part of other workers. Reliability, as evaluated by the one-month test-retest Spearman-Brown split-half coefficient was .91 [19].

Mental health was assessed by the 12-item version of the General Health Questionnaire (GHQ-12) [37] in its Italian version [38]. Using the Likert scoring method from 1 to 4, we created a “Psychological problems” variable, ranging from 12 to 48. In this study, the reliability coefficient (Cronbach’s alpha) of GHQ-12 was .87.

Job stress was evaluated by Karasek’s demand/control/support model [39]. The 17-item questionnaire consisted of 3 scales termed ‘psychological job demand’, ‘job control or decision latitude’ and ‘workplace social support’. The ‘demand’ scale was the sum of 5 items (e.g. “Do you have to work very fast in your job?”); the ‘control’ scale was the sum of 6 items (e.g. “Do you have the opportunity to learn new things in your work?”); and the ‘support’ scale was the sum of 6 items (e.g. “There is a calm and pleasant atmosphere where I work”). Items were scored using a 4-point Likert scale in which the first two scales were graded from 1 = never to 4 = often, while the third scale (support) was graded from 1 = strong disagreement to 4 = strong agreement. The reliability of the scales in this sample was .80 for demand, .66 for control and .84 for support, not unlike the values observed in the original validation study [40].

The perceived level of justice at work was assessed by the Justice Measurement questionnaire [41] in its Italian version [42]. This is an indirect measure of fairness that considers different components (procedural, distributive, interpersonal and informational) of organizational justice. The questionnaire consists of 20 items, graded on a 5-point Likert scale ranging from 1 = to a small extent to 5 = to a large extent. (e.g.“To what extent are procedures applied consistently?”; “To what extent does your superior treat you with respect?”). In our sample, the reliability of the questionnaire, as measured by Cronbach’s alpha, was .92.

### Statistics

Analyses began with basic descriptive statistics on the sample and the consequences of reported events, and crude estimates of event rates. For each respective year, comparisons between groups were made by chi square and one-way ANOVA statistics.

Logistic regression analysis was used to study the association between individual and occupational variables and the reporting of aggression in the entire period from 2005 to 2009.

In the 2009 survey, the association of psychosocial variables with violence was also studied by logistic regression analysis. Univariate models included each job stress variable (demand, control and social support), organizational justice, and psychological distress. Significant associations were then studied using multivariate models adjusted for age, gender, job and department type. Odds ratios (OR) and 95 % confidence intervals (CI) were calculated.

The statistical package PASW/SPSS 15.0 was used for the analysis.

## Results

1,166 out of a total of 1,455 workers (387 male, 33.2 %, 779 female, 66.8 %) took part in the survey. The average participation rate of 80.1 % ranged from a minimum of 71 % in 2007 to a maximum of 94 % in 2009. Table 1 illustrates the characteristics of the sample and the distribution of workers in the various categories.

**Table 1:** Characteristics of the observed sample

	**2005**	**2007**	**2009**	***p***
N. of workers (% response rate)	367 (75 %)	346 (71 %)	453 (94.2 %)	92.4 (<.001)^2^
Male workers N (%)	145 (39.5 %)	115 (33.2 %)	127 (28.0 %)	12.0 (<.002) ^2^
Female workers N (%)	222 (60.5)	231 (66.8)	326 (72.0 %)	
Age, mean+s.d. (years)	43.6+7.8	43.4+ 8.9	42.4+ 9.6	.138 (n.s.)^1^
Physicians, N (%)	58 (15.8)	69 (19.9)	59 (13.0)	7.01 (<.04) ^2^
Nurses, N (%)	207(56.4)	106 (30.6)	263 (58.1)	69.5 (<.0001) ^2^
Other^3^, N (%)	102 (27.8)	171 (49.4)	131 (28.9)	47.5(<.0001) ^2^
Psychiatry	28 (7.6)	40 (11.6)	42 (9.3)	17.7 (<.03) ^2^
A & E	60 (16.3)	49 (14.2)	66 (14.6)	
Inpatient wards	121 (33,0)	112 (32,4)	156 (34,4)	
Outpatient	74 (20,2)	73 (21,1)	60 (13,2)	
Laboratory and services	84 (22,9)	72 (20,8)	129 (28,5)	

n.s.: not significant.

(^1^): one-way analysis of variance; (^2^) Pearson’s chi square.

(^3^): this category includes: laboratory technicians, radiology technicians, physiotherapists, ancillary personnel, blue collar workers and clerks.

Across the entire study period, a total of 107 workers (9.2 %) reported suffering a physical aggression in the twelve-month period preceding the survey; 101 reported suffering threats and 229 (19.6 %) reported being the victims of verbal aggression. In 2009, 25 workers (5.5 %) reported being the victims of harassing behavior (Table 2). The prevalence of physical aggression on health workers remained fairly stable during the period under review (8.2 % in 2005, 9.2 % in 2007, 9.9 % in 2009; Pearson’s chi square *p* = .685).

**Table 2:** Prevalence of violence

	**2005**	**2007**	**2009**	***p***
N. of workers	367	346	453	
ONE-YEAR PREVALENCE				
Physical aggression, N (%)	30 (8.2)	32 (9.2)	45 (9.9)	.685 (n.s.)^1^
Threats, N (%)	44 (12.0)	96 (27.7)	57 (12.6)	.006 (n.s.) ^1^
Non-physical aggression, N (%)	72 (19.6)		61 (13.5)	5.6 (<.02) ^1^
Harassment, N (%)	n.a.	n.a.	25 (5.5)	
LIFE PREVALENCE				
Physical aggression, N (%)	91 (24.8)	n.a.	120 (26.5)	.30 (n.s.) ^1^
Threats, N (%)	123 (33.5)	n.a.	114 (25.2)	6.9 (<.01) ^1^
Non-physical aggression, N (%)	156 (42.5)	n.a.	144 (31.8)	10.0 (<0.01) ^1^

n.a.: not applicable.

n.s.: not significant.

(^1^): Pearson’s chi square.

Physical aggression and threats directed towards males were slightly more frequent than towards females, although a statistically significant difference was observed only for threats (OR 1.66 95%CI 1.08-2.54 in males vs. females). Females were the principal victims of harassment, but the difference in this case between males and females failed to reach significant levels (Table 3). Gender differences tended to disappear and were no longer significant after correction for job type (Table 4).

**Table 3:** Relative risk of violence at work in the different categories of health workers from 2005 to 2009

	**Physical aggression (N = 107)**	**Threat^1^(N = 101)**	**Verbal violence(N = 229)**	**Harassment^2^(N = 25)**
Male	1.00(0.74-1.70)	1.66 (1.08-2.54)*	1.02 (0.75-1.39)	0.99 (0.41-2.45)
Female	1	1	1	1
Physician	2.20 (1.16-4.16)*	4.32 (1.94-9.64)***	1.27 (0.84-1.94)	2.80 (0.90-8.75)
Nurse	2.36 (1.42-3. 92)**	4.03 (2.04-7.98)***	0.92 (0.67-1.28)	0.99 (0.37-2.72)
Other workers	1	1	1	1
Psychiatry	22.69 (9.75-52.82)***	27.79 (11.45-67.49)***	3.50 (2.10-5.84)***	2.61 (0.67-10.21)
A&E	5.12 (2.12-12.39)***	7.65 (3.21-18.23)***	1.99 (1.23-3.22)***	1.60 (0.42-6.17)
Medical & surgical wards	3.44 (1.49-7.93)**	2.54 (1.06-6.06)*	1.37 (0.90-2.08)	1.70 (0.56-5.10)
Outpatient	1.81 (0.66-4.93)	2.89 (1.11-7.55)*	1.25 (0.76-2.04)	0.85 (0.16-4.54)
Services	1	1	1	1

Legenda: (^1^) data not collected in 2007; (^2^) data not collected in 2005 and 2007.

(*):p < .05; (**)p < .01; (***) p < .001.

Univariate logistic regression models, odds ratios (ORs) and 95 % confidence interval (CI95%).

**Table 4:** Multivariate Logistic regression models, odds ratios (ORs) and 95 % confidence interval (CI95%) corrected for age, gender, job and department

	**Physical aggression (N = 107)**	**Threat^1^(N = 101)**	**Verbal violence(N = 229)**	**Harassment^2^(N = 25)**
Male	1.00 (0.62-1.63))	1.49 (0.90-2.47)	0.93 (0.66-1.32)	0.77(0.30-2.01)
Female	1	1	1	1
Physician	0.99 (0.47-2.11)	1.49 (0.54-4.10)	0.86 (0.52-1.41)	1.69 (0.42-6.90)
Nurse	1.25 (0.69-2.27)	1.63 (0.65-4.08)	0.63 (0.42-0.94)*	0.52 (0.14-1.98)
Other workers	1	1	1	1
Psychiatry	19.80 (7.82-50.11)***	21.69 (6.86-68.56)***	4.55 (2.49-8.29)***	3.71(0.70-19.55)
A&E	4.29 (1.61-11.40)***	6.03 (1.98-18.39)***	2.73 (1.55-4.84)***	1.89 (0.37-9.69)
Medical & surgical wards	2.75 (1.09-6.96)**	2.07 (0.69-6.28)	1.91 (1.15-3.18)*	2.54 (0.61-10.62)
Outpatient	1.57 (0.54-4.59)	2.70 (0.83-8.75)	1.70 (0.93-2.92)	1.13 (0.18-7.06)
Services	1	1	1	1

Legenda: (^1^) data not collected in 2007; (^2^) data not collected in 2005 and 2007.

(*):p < .05; (**)p < .01; (***) p < .001.

Nurses and doctors were the professional groups most exposed to physical aggression and threats. A comparison with all other workers indicated that both nurses and physicians were at greater risk of physical and non-physical violence (Table 3). However, after adjustment for department type, the odds ratios were found to be lower and no longer significant (Table 4).

Results showed that workers in psychiatric and emergency services are those at greatest risk of physical aggression, since about half of all violence of this kind is concentrated in these health care areas. Psychiatric service workers reported 40 episodes of physical violence (annual rate of aggression for employed persons = 36.4 %). If the risk of a worker engaged in business services (laboratory, radiology, offices) is taken to be one, the risk of physical violence for employees in mental health services is twenty-two times higher (OR 22.7, 95%CI 9.7-52.8) and the risk of being threatened as much as twenty-seven times higher (OR 27.8 95%CI 11.5-67.5). Twenty cases were reported (annual rate = 11.4 %) in the Accident and Emergency Department. Staff in accident and emergency departments were at a significantly higher risk of violence (OR 5.1, 95%CI 2.1-12.4 for physical aggression, OR 7.6, 95%CI 3.2-18.2 for threats). The remaining episodes of violence were reported by workers in medical and surgical wards (31 cases, 8,0 %), in outpatients (9 cases, 4.3 %) and services (7 cases, 2.5 %). Workers in surgical and medical wards reported a higher risk of violence (OR 3.4, CI95% 1.5-7.9 for physical aggression, OR 2.5 CI95% 1.1-6.1 for threats) than service workers (Table 3). These associations were slightly less apparent after correction for confounders, while increases in the odds ratios continued to be highly significant (Table 4).

In 2009, 45 workers (10 %) reported that they had been physically assaulted in the previous 12-month period, and 75 more workers reported experiencing at least one upsetting episode of physical aggression in the past; overall, 120 workers (26.5 %) reported at least one incident involving physical aggression during their professional lives (Table 5). The perpetrators of this violence were mainly patients (82, 68.3 %) or visitors (26, 21.7 %). In a minority of cases the assailant was a colleague (9, 7.5 %). Perpetrators were usually male (75.8 %).

**Table 5:** Characteristics and consequences of upsetting aggression reported in the 2009 survey

	**PHYSICAL AGGRESSION**	**NON PHYSICAL AGGRESSION**
**Reported aggression, N (%)**	120 (26.5)	144 (31.8)
**Type of aggressor**		
“External”		
Patient N (%)	82 (68.3)	52 (36.1)
Patient’s relative or friends N (%)	26 (21.7)	35 (24.3)
Other people N (%)	3 (2.5)	7 (4.9)
“Internal”		
Colleague, staff N (%)	9 (7.5)	50 (44.7)
**Gender of aggressor**		
Male	91 (75.8)	104 (72.2)
Female	29 (24.2)	40 (27.8)
**Age of aggressor**		
<29	28 (23.3)	12 (8.3)
30-39	39 (32.5)	32 (22.2)
40-49	26 (21.7)	42 (29.2)
50-59	14 (11.7)	38 (26.4)
60+	13 (10.8)	20 (13.9)
**Result of aggression**		
Fear	27 (22.5)	22 (15.3)
Anger	40 (33.3)	65 (45.1)
Distress	18 (15.0)	17 (11.8)
Anxiety	21 (17.5)	35 (24.3)
Humiliation	8 (6.7)	15 (10.4)
Guilt	6 (5.0)	6 (4.2)
Disappointment	19 (15.8)	40 (27.8)
Helplessness	27 (22.5)	27 (18.8)
Physical injury	11 (9.2)	-
No reaction	31 (25.8)	37 (25.7)
Desire for revenge	11 (9.2)	22 (15.3)
Feeling of being wrong	20 (16.7)	35 (24.3)
Intention to change place of study/work	25 (20.8)	52 (36.1)
Intention to change behavior	27 (22.5)	23 (16.0)
**Reporting**		
The aggression was reported to superiors	58 (48.3)	52 (36.1)
The aggression was reported to friends or relatives		28 (19.4)
The aggression was reported to a physician at A&E	4 (3.3)	1 (0.69)
The aggression was reported to the police	7 (5.8)	
The aggression was not reported	51 (42.5)	63 (43.8)

Non-physical aggression was more frequent than the physical type; a total of 144 workers (31.8 %) reported having been exposed to non-physical violence and described their experience. Patients (52, 36 %), their relatives and friends (35, 24 %) were responsible for more than half of these episodes; most of the remaining cases of violence were perpetrated by colleagues or superiors (50, 45 %).

The consequences of physical and non-physical violence included anger, disappointment, anxiety, distress and the intention to move to another place of work or to perform professional duties in a different way.

The occurrence of workplace violence was severely under-reported to health or police authorities. Even though we asked for a description of the worst episode of violence experienced, only about half of these incidents had been reported either to a colleague or a friend. Only in exceptional cases was a report made to the Accident Department or the police (Table 5).

Logistic regression analysis (Table 6) showed that work-related stress measured by the demand/control/support model and by organizational justice and psychological disorders appeared to be associated with non-physical violence after adjustment for age, gender, job type and department type. In particular, high demand, low support, low organizational justice, and high psychological disorders scores were associated with non-physical violence (p < .001). Workers reporting internal, worker-on-worker verbal violence manifested low control (p < .05), low support (p < .001), low justice (p < .001), and high psychological disorders (p < .001) in comparison with other workers (Table 6).

**Table 6:** Association of psychosocial variables with violence reported in the 2009 survey

**Variable**		**Physical aggression, whole life (N = 120)**	**Physical aggression, 2009 (N = 45)**	**Non physical aggression, whole life (N = 144)**	**Non physical aggression, 2009 (N = 61)**	**Internal, non physical aggression, whole life (N = 53)**
Demand	Un.	1.06 (0.99-1.14)	1.07 (0.97-1.18)	1.18 (1.11-1.26)***	1.14 (1.04-1.24)***	1.00 (0.89-1.11)
	Ad.	1.07 (0.99-1.16)	1.12 (0.99-1.27)	1.17 (1.10-1.26)***	1.14 (1.03-1.26)***	1.02 (0.90-1.15)
Control	Un.	1.01 (0.94-1.09)	0.98 (0.87-1.09)	1.00 (0.93-1.08)	0.96 (0.87-1.05)	0.83 (0.73-0.95)***
	Ad.	0.97 (0.89-1.06)	0.90 (0.79-1.03)	0.98 (0.90-1.06)	0.94 (0.85-1.04)	0.85 (0.74-0.98)*
Support	Un.	0.95 (0.89-1.02)	0.93 (0.85-1.02)	0.86 (0.81-0.92)***	0.85 (0.79-0.92)***	0.80 (0.71-0.89)***
	Ad.	0.95 (0.89-1.02)	0.91 (0.82-1.00)	0.85 (0.80-0.91)***	0.85 (0.78-0.92)***	0.79 (0.69-0.90)***
Justice	Un.	0.98 (0.96-0.99)*	0.98 (0.95-0.99)*	0.97 (0.95-0.98)***	0.97 (0.95-0.99)***	0.95 (0.93-0.98)***
	Ad.	0.98 (0.97-1.00)	0.98 (0.95-0.99)*	0.97 (0.95-0.98)***	0.97 (0.95-0.98)***	0.96 (0.93-0.99)***
Psychological disorders	Un.	1.03 (0.99-1.07)	1.00 (0.94-1.06)	1.11 (1.07-1.16)***	1.12 (1.06-1.17)***	1.11 (1.05-1.18)***
	Ad.	1.02 (0.97-1.07)	1.00 (0.94-1.08)	1.11 (1.06-1.16)***	1.11 (1.05-1.16)***	1.12 (1.04-1.20)***

Logistic regression analysis, Odds ratios (OR) and 95 % CIs (unadjusted and adjusted for age, gender, job, and department). (*):p < .05; (**)p < .01; (***) p < .001.

## Discussion

A significant proportion of our sample reported experiences of workplace violence. We found that employees who experienced verbal violence in clinical settings had lower levels of perceived organizational justice and social support, higher levels of work-related stress and higher psychological problem scores than other HCWs. An important finding that emerged from this study is that isolated workers (with low social support) are exposed to violence, and that employees who experience violence are psychologically distressed. They manifest high levels of job strain and perceive their health care facility to be unfair. We also observed an interesting association between lifetime exposure to internal non-physical violence and low job control. Perceived control is a measure of power in the organization and it is significant that verbal violence, which is an abuse of power, is exerted by colleagues on workers with less authority. The cross-sectional nature of our study prevents us from indicating a definite cause for the association between violence and psychosocial variables. However, the association of workplace violence with high demand and psychological disorders, and the protective role of job control, social support and organizational justice suggest that preventive programs should target these variables.

The repercussions resulting from violence in the workplace are important as they can lead to a deterioration in staff health. A systematic review of studies on aggression showed that despite differences in countries, cultures, research designs and settings, the responses of health care workers to patient aggression are similar and include immediate responses such as frustration, fear, anger or anxiety [9,43]. These responses may also extend to become addiction [44], burnout [45], post-traumatic stress disorder, guilt, self-blame, and shame [46], an intention to quit nursing and an intention to change institution [11]. These psychological effects can persist for months or years after the original event occurred [9]. Our study confirms that non-physical violence is associated with high work-related stress, high psychological distress and the perception of unfairness on the part of the health care facility/organization. The most likely interpretation of this result is that violence causes stress and a perception of injustice, but the cross-sectional nature of the study does not exclude the theory (suggested by some authors [14,22]) that stressed workers are more likely to be victims of violence.

As expected, nurses were the category most exposed to physical violence. Nurses are more likely to encounter aggressive behavior because of the amount of time they spend providing direct patient care. Studies show that the chances of suffering physical violence are 7.2 and 9.0 times greater for healthcare workers with moderate and high patient contact, respectively, than for those with little or no contact [47]. In our sample, the percentage of nurses reporting one episode of physical aggression over the previous year (11.5 %) roughly corresponds to the average one-year aggression rate found by Hodgson et al. in 142 American hospitals [8], and by Gerberich et al. [9] in the Minnesota Nurses’ study on 6,300 randomly selected nurses. Our study shows that physicians are also at a significant risk of aggression. Contrary to what is normally seen, in some cases the rate of aggression was higher for doctors than for nurses. This may be due either to the role and decision-making power of doctors, especially in some high-risk medical services, or to the fact that nurses tend to report incidents less frequently than doctors.

Respondents from the psychiatric department experienced the highest overall level of patient-initiated aggression. One out of three workers reported being assaulted in the previous year, and almost three quarters of these workers had experienced physical violence during their working life. The A&E (accident and emergency) department also seemed to be a particular target for physical and verbal aggression. More than half of all the reported cases of physical and verbal violence had occurred in these high-risk departments. A high prevalence of violence in psychiatric settings has been observed in earlier studies [48] and confirmed in subsequent ones [12,13,27,49,50]. Both patient complaints originating from environmental conditions and poor communication [51] and staff-related factors, such as low work experience [12,48], low general health [50], anxiety [13] and low job satisfaction [47,48], are associated with aggression. The incidence of workplace violence in the emergency department has been well documented in numerous published studies [49-55]. Besides nurses and physicians, other professionals working in psychiatric [27,56] and emergency departments [57] are also at risk.

Our study has several limitations. Firstly, since our investigation was limited to a single health care district, we cannot extend our findings to all Italian health care services. However, our results are in agreement with the literature, and we have no evidence to suggest that the situation is different in other health care facilities.

Secondly, the survey was a retrospective one, with the usual limitations of inaccurate recall of past events and of possible contamination by current events. However, the repeated measurement of aggression rates over time and their relative stability indicate that the phenomenon exists. The present study had a high participation rate compared to other similar studies on this topic, thereby increasing our confidence in the results.

Thirdly, this study was limited to an exploration of abuse from a worker perspective; no attempt was made to validate the episodes reported. In fact, no objective criteria for misconduct were specified, and the findings presented here should be considered hypothetical. What workers perceived as misconduct may not constitute misconduct in an objectively ethical or legal sense. However, it is the perception of the event and not the event itself that may have the greatest impact on the individual [58]. It has also been suggested that it may be in the interest of some professionals and labor unions to highlight rates of violence in the workplace as a way of enhancing their role in protecting workers [10]. By informing workers of the opportunity to fill in a VIF during their periodical medical examination, we chose a method that was unlikely to be influenced by the interests of consultants or unions.

Fourthly, the present study relied on self-reported measurements, which could lead to problems associated with an inflation of the strength of relationships and with the common method variance. However, to minimize problems with self-reports we used well-known validated questionnaires that have shown good reliability. Moreover, although we attempted to exclude all possible confounders, we cannot rule out the existence of residual confounding.

Our study has some strong points. The collection of data on the part of a physician who was not a direct employee of the health care facility, but who had a direct knowledge of workplaces and had had a long-lasting relationship with workers, increased the response rate and reduced the likelihood of inappropriate responses. Furthermore, this method could encourage workers to take part in prevention by suggesting possible remedies for workplace violence.

We fully agree with the suggestion of the Italian Ministry of Health that an assessment of violence is necessary in all health care organizations. No paramount episode of violence had been reported to the occupational physician before workers began completing the questionnaires.

## Conclusion

In conclusion, our study corroborates previous reports of frequent physical and verbal aggression towards HCWs. In a standard public health service, nurses and doctors working in psychiatric services and accident and emergency departments are the most exposed to attacks. Abused workers suffer higher job stress, greater psychological distress, have a greater sense of injustice and lower social support than other workers. It is likely that a mutual increase occurs between workplace violence and psychosocial problems.

Violence at work is a hidden phenomenon and in most Italian health care facilities there is no policy for prevention. Our study indicates that the problem exists and that prevention is essential.

Most of the physical aggression and a significant proportion of the verbal aggression experienced by clinical staff are the result of patient interactions and generally regard clinical issues arising from patient care. Training workers in good working practices and alternative methods of resolving disputes is generally seen as the way to reduce the likelihood of this type of aggression [8], especially if it is accompanied by organizational and environmental safety measures. Teamwork and a supportive workplace have also been shown to mitigate this type of workplace violence [59]. The use of interdisciplinary multi-level prevention programs that have proven effective in a small private health care facility [36] would also be beneficial in public health care settings.

Our experience indicates, however, that a significant proportion of the violence encountered in the clinical setting is perpetrated by other health care workers and it is this latter form of violence that is most closely linked to psychosocial variables. Traditional methods, such as the development of personal safety skills and de-escalation techniques, or institutional policies and environmental design may not be sufficient to prevent this kind of violent behavior. These should therefore be integrated with specific intervention targeted at root causes such as conflict in the workplace. The association of violence with psychosocial variables indicates the need for far-reaching changes in health care organization that should include decision-making procedures, work climate and support, and relations between workers. Counteracting violence requires strong commitment on the part of both workers and management.

## Competing interests

The authors declare that they have no competing interests.

## Authors’ contributions

NM devised the study and collected and processed the data; TH revised the statistical analyses and helped to draft the manuscript. All authors read and approved the final manuscript.

## Funding

No funding was received by the first author for the study or preparation of the manuscript. Tarja Heponiemi was funded by the Academy of Finland (project no: 128002).

## Pre-publication history

The pre-publication history for this paper can be accessed here:

http://www.biomedcentral.com/1472-6963/12/108/prepub
